# Elucidating the Complex Interactions between Stress and Epileptogenic Pathways

**DOI:** 10.1155/2011/461263

**Published:** 2011-03-20

**Authors:** Aaron R. Friedman, Luisa P. Cacheaux, Sebastian Ivens, Daniela Kaufer

**Affiliations:** ^1^Department of Integrative Biology, University of California-Berkeley, Berkeley, CA 94720-3140, USA; ^2^Institute of Neurophysiology, Charité University Medicine, 10117 Berlin, Germany; ^3^Department of Psychiatry and Psychotherapy, Charité University Medicine, 10117 Berlin, Germany; ^4^Helen Wills Neuroscience Institute, University of California-Berkeley, Berkeley, CA 94720-3140, USA

## Abstract

Clinical and experimental data suggest that stress contributes to the pathology of epilepsy. We review mechanisms by which stress, primarily via stress hormones, may exacerbate epilepsy, focusing on the intersection between stress-induced pathways and the progression of pathological events that occur before, during, and after the onset of epileptogenesis. In addition to this temporal nuance, we discuss other complexities in stress-epilepsy interactions, including the role of blood-brain barrier dysfunction, neuron-glia interactions, and inflammatory/cytokine pathways that may be protective or damaging depending on context. We advocate the use of global analytical tools, such as microarray, in support of a shift away from a narrow focus on seizures and towards profiling the complex, early process of epileptogenesis, in which multiple pathways may interact to dictate the ultimate onset of chronic, recurring seizures.

## 1. Introduction

In clinical studies of epilepsy patients, stress is the most frequently self-reported trigger of seizures—higher than other precipitants such as sleep deprivation, fatigue, diet, or even missed medication [1–3]. In prospective study, increased severity of self-reported stress/anxiety also correlates with increased risk of subsequent seizure [4]. These studies, derived from the first-hand experience of those who suffer from epilepsy, set the stage for a wealth of experimental data indicating that stress may impact and exacerbate epilepsy in at least four contexts: (1) life stress, particularly early life stress, may create a vulnerability for the incidence of epilepsy; (2) stress may play a role in the etiology of symptomatic epilepsy by exacerbating the causal event, such as traumatic brain injury, stroke, or status epilepticus; (3) stress may play a role in the process of epileptogenesis—the “silent period” that follows initial injury and is characterized by progressive cellular and network changes thought to underlie the ultimate onset of chronic seizures; (4) stress may increase the frequency or severity of seizures after epilepsy onset. In this paper, we focus on the molecular pathways by which stress and epilepsy may converge. We emphasize the nuance and complexity of these pathways, noting that the role of particular molecules can vary from neuroprotective to destructive, depending on context. To highlight this complexity, we discuss the signaling pathways that are initiated following blood-brain barrier dysfunction and emphasize neural-glia interactions. Finally, in light of the complex interplay of pathways that affect epilepsy, we advocate strategies for “global” characterization of epilepsy pathology to complement the single pathway investigation typically favored by inference-guided experimentation.

## 2. Mechanisms by Which Stress May Create Vulnerability to Epilepsy

In addition to the immediate physiological “stress response,” stressful incidents, particularly early in life, can cause long-term changes in the organism that create vulnerability for a variety of diseases [5, 6]. This concept of stress-induced vulnerability has not been frequently applied to epilepsy, despite evidence that many persistent changes induced by stress are likely to affect mechanisms of epilepsy. Perhaps most importantly, early life stress can cause long-lasting alterations in the regulation of the hypothalamic-pituitary adrenal (HPA) axis [7], which controls the release of stress hormones (glucocorticoids; GCs). These alterations, which are effected by cognitive mechanisms (neural plasticity in reinforcing stress-responsive networks [8, 9]) and genetic transcriptional mechanisms (classical and epigenetic regulation of genes controlling the HPA axis [10, 11]), lead to adult animals that have an impaired stress response to aversive stimuli, including increase in stress hormone release and impairment of HPA negative feedback [12]. Thus, all aspects of the stress response that may directly exacerbate epilepsy (described in subsequent sections) are likely to be particularly potent in individuals that have experienced early life stress. For example, early life stress affects adult induction of immune and inflammatory pathways [13, 14], which have been implicated in neural damage in epilepsy. Similarly, early life stress decreases the expression of brain-derived neurotrophic factor (BDNF) in the adult brain [15–17], which is a critical mediator of neuroprotection across epilepsy models.

Early life stress may also have a profound impact on the development of white matter in the brain. Preliminary work in our lab and others indicates that stress may increase or decrease myelination, depending on developmental stage and other unknown factors (unpublished data and [18]). These paradoxical findings are echoed by the literature showing that GCs induce *in vitro* oligodendrocyte precursor cells (OPCs) to differentiate into mature oligodendrocytes [19–21] and promote oligodendrocyte survival [22], yet total removal of GCs by adrenalectomy results in hypermyelination [23] while prenatal GC treatment delays myelination in sheep [24]. If early life stress does result in delayed and/or hypomyelination, it would constitute a startling and underappreciated similarity to a variety of seizure syndromes. Delayed myelination is a hallmark of infantile spasms [25] and other seizure disorders [26], and several genetic hypomyelination disorders or manipulations include severe seizure symptoms [27, 28]. In these models, treatment is associated with white matter recovery: amino acid supplement of patients with a serine biosynthesis disorder resulted in restoration of white matter and major seizure reduction [29, 30]. Pharmacologically (L-allylglycine, bicuculline, and kainic acid) or electrically induced seizures also cause demyelination [31, 32], and alterations of white matter have been associated with both symptomatic and idiopathic epilepsy [33–35] and with hippocampal sclerosis [36]. Indeed, while glia have received a surge of recent interest for causal roles in epilepsy, this attention has focused almost exclusively on astrocytes. Possible roles for oligodendrocytes remain largely uninvestigated.

Direct investigation of early life stress on subsequent epilepsy is sparse, but there have been at least a few studies in rodents. One study subjected pups to maternal separation (MS) or normal rearing and then induced status epilepticus (SE) by lithium-pilocarpine at P16 and assessed subsequent advent of behavioral seizures in adulthood. Only one normally reared rat showed adult spontaneous recurrent seizures (SRS), whereas all 8 rats from the MS group developed SRS [37], though it is not clear if this difference can be attributed to a persistent “vulnerability” created by MS or to a more immediate effect of the MS stress on severity of induced SE. Another group subjected rats to MS or mild handling and assessed seizure induction by amygdala kindling subsequently in adulthood. MS rats required significantly less stimulation for seizure induction [38]. Gendered analysis indicates that this effect may only hold true for female rats [39]—an interesting finding given the well-known effects of sex hormones both on stress response and on epilepsy [9, 40–42]. A similar study showed that chronic GC supplement in adult adrenalectomized rats also accelerated the rate of amygdala kindling [43], indicating that interactions between GCs and seizure threshold may be generalized outside of the early life period. 

The limited available direct evidence, as well as general observations of persistent changes mediated by early life stress, indicates that it could cause a life-long vulnerability for subsequent epilepsy. Given that the factors that govern whether or not epileptogenesis occurs after traumatic injury are poorly understood, the role of early life stress vulnerability deserves more in-depth study.

## 3. Mechanisms by Which Stress May Exacerbate Etiological Incidents

The most common form of symptomatic epilepsy involves a precipitating traumatic incident—an initial prolonged seizure (SE), stroke, traumatic brain injury, or infection/fever—that is followed by onset of epilepsy after a delay of months to years. A wealth of evidence indicates that damage suffered during such incidents and possibly also the induction of repair mechanisms constitute the first steps of epileptogenesis. Can activation of stress pathways during etiological incidents exacerbate damage or otherwise contribute to the proximate steps of epileptogenesis?

One of the common occurrences across different types of precipitating incidents is immediate neurological injury and cell death. GCs exacerbate such neural injury. For example, viral vector blockade of glucocorticoid receptors (GR) during kainic acid (KA) treatment (used to induce SE and associated excitotoxic cell death) significantly reduced the size of the ensuing hippocampal lesion and also significantly reduced cell death in KA-treated neural cell culture cotreated with GCs [44]. The damaging effects of GCs appear to be at least partially dependent on their downregulation of BDNF, as exogenous BDNF also attenuates the *in vitro* cell death. GC induction of proinflammatory pathways (discussed in Section 4) also plays a major role by leading to excitotoxic cell death [45]. Similarly, stress treatment prior to stroke (via the middle cerebral artery occlusion model) increases levels of pro-inflammatory TNF-*α* and Il-1*β*, causing more extensive cell death in the infarct [46, 47]. 

Breakdown of the blood-brain barrier (BBB) is also common across etiological incidents. Research in our lab and in our collaborator's has shown that BBB disruption allows serum albumin to enter the brain and activate the transforming growth factor beta receptor (TGF-*β*R) signaling pathway in astrocytes, ultimately inducing epileptiform activity and spontaneous seizures. Blockade of the TGF-*β*R prevents albumin-induced signaling, epileptiform activity, and reduces seizures detected by EEG monitoring ([48–50] and unpublished data). Interestingly, stress also disrupts the BBB [51–53] and thus may directly contribute to postinjury BBB leakiness, likely through induction of pro-inflammatory pathways [54].

## 4. Mechanisms by Which Stress May Contribute to Epileptogenesis

Beyond the proximate precipitating incident, the process of epileptogenesis occurs over a period of weeks to years and is marked by a somewhat stereotypical progression of restructuring events that precede the onset of chronic spontaneous seizures [55, 56]. The role of astrocytes in this process has come to be one of the most studied frontiers in epilepsy research, due to the effects of activated astrocytes and gliosis on regulating excitability via extracellular ions and neurotransmitters, and to the association of glial scars with hippocampal sclerosis [57, 58]. Pro-inflammatory cytokine pathways are common mediators of astrocyte activation and epileptogenesis across epilepsy models. For example, albumin activation of the TGF-*β* pathway in astrocytes leads to the induction of pro-inflammatory and cytokine pathways including NF-*κ*B [48]. Similarly, pilocarpine-induced SE causes an increase in leukocyte adhesion molecules and local leukocyte recruitment, a critical first step in the induction of the pro-inflammatory immune response [59]. In both cases, blockade of this initial pro-inflammatory event prevents subsequent onset of epileptic activity. While GCs are generally thought of as anti-inflammatory, and indeed often used as therapeutic peripheral anti-inflammatory agents, they actually have pro-inflammatory roles within brain [60]. Indeed, stress increases the expression or activity of a number of mediators of inflammation in the brain, including NF-*κ*B, TNF-*α*, IL-1*α*, IL-1*β*, prostaglandins, and free radicals such as NO [45, 61–63] via both catecholamines and GCs [60, 64]. Thus, stress would be expected to enhance pro-inflammatory pathways that are major aspects of epileptogenesis.

Aberrant neurogenesis in the hippocampus is also a hallmark of epileptogenesis [65, 66], including the ectopic migration of new neurons into the hilus. Stress and GCs influence the proliferation, differentiation, and survival of neural stem cells in the hippocampus [67–69]. Generally, stress decreases neurogenesis at both proliferation and survival stages [69], but also decreases the percentage of precursor cells that adopt a neural cell fate (unpublished data). It is unknown how or if stress effects on neurogenesis may interact with aberrant neurogenesis during epileptogenesis.

## 5. Mechanisms by Which Stress May Exacerbate the Frequency or Severity of Seizures

After the progression of epileptogenesis and the onset of epilepsy, patients experience spontaneous recurrent seizures that vary in frequency and severity. As mentioned in the introduction of this paper, stress is the major self-reported precipitant affecting seizure frequency. In support of these clinical studies, stress pathways have been shown to promote neural activity in a variety of ways, suggesting that stress may directly contribute to the hyperexcitability that causes spontaneous seizures. Corticotropin-releasing hormone (CRH)—which is released in the brain as the first step of the stress hormone response—causes an increase in neural discharge and modulates glutamatergic transmission [70–72]. While CRH acts directly on a variety of neural receptors [71, 73], it also ultimately induces release of GCs from the adrenal glands. GCs themselves increase the release of excitatory glutamate [74], while stress paradigms similarly induce an increase in extracellular glutamate and aspartate [75]. Excitatory actions of GCs can be mediated by fast-acting protein mechanisms [76–78] as well as the classical delayed (transcriptional) effects of GR, which have been shown to modulate calcium currents in particular [79, 80].

## 6. Complexity in Epileptogenic Pathways and Experimental Implications

We see from the above that stress pathways converge with a variety of other signaling pathways associated with epilepsy, including regulators of excitotoxic cell death, myelination, inflammation, astrocytic activation, and neurogenesis. The nature of this interaction depends on the timing of stress relative to the progression of epilepsy. However, a variety of other variable factors make these pathway interactions quite complex. Firstly, it should be noted that many events associated with epileptogenesis, such as inflammation, gliosis, and neurogenesis, are frequently assumed to be pathological. Equally plausible in many cases is that these pathways may represent (failed) attempts of neuroprotection and recovery. Stress itself is often conceptualized as having an “inverted U-shaped” effect on a given task or output, with extremely low or high amounts of GCs being “detrimental” but moderate amounts being “beneficial.” Furthermore, it is widely recognized that the effects of stress may vary depending on task or context. For example, early life stress is detrimental by many metrics, but may also lead to a blunting of inflammatory response that is protective in terms of epilepsy vulnerability [60]. This type of nuanced analysis must be applied when considering the effect of stress on epilepsy (protective and detrimental effects of stress on epilepsy are reviewed in depth by [81]); it would also be well applied to epileptic pathways in themselves. Inflammation, reactive astrocytes, and neurogenesis in particular have been alternately described as protective or pathological. The specific effects of these mechanisms may vary depending on severity of injury (i.e., protective after mild precipitating brain trauma, but overexpressed and damaging after severe brain injury) or on the specific stage of epileptogenesis.

Similarly, it is important to consider that most of these mechanisms are investigated in the context of “strong inference” type experimentation, wherein a specific pathway is genetically or pharmacologically manipulated, and specific outputs such as cell death or seizure onset are interpreted as markers of pathology. In taking a step back to a more global view of epilepsy, we emphasize that a large number of molecular mechanisms are in play at any given moment, interacting in complex ways. What is the net output, for example, when pathways known to be neuroprotective, and others known to cause neural damage, are induced at the same time?

**Figure 1:**
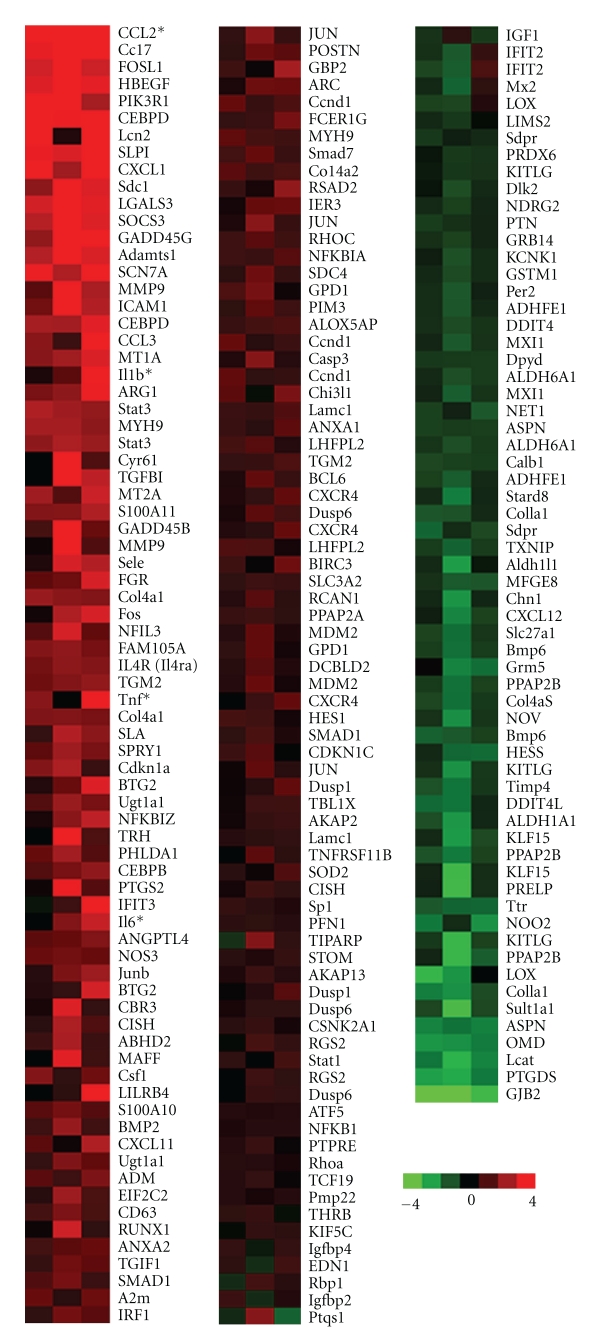
Transcriptional analysis of GC-responsive genes that are modulated by albumin treatment. Arrays from three animals that were sacrificed 24 hours after albumin treatment [48] were reanalyzed to identify genes that are modulated by both stress and the model of albumin-induced epileptogenesis. *Genes mentioned in text.

To address such complexity in epileptogenic mechanisms, we advocate the use of global analytical tools such as microarrays. While microarrays are frequently used as a discovery tool, they may also be used in a much more targeted fashion to characterize global events surrounding a specific mechanism. For example, we used microarrays to characterize the transcriptional profile that follows albumin binding to TGF-*β*Rs, showing that this proximal event triggers a cascade that is highly similar to TGF-*β* signaling induced by the endogenous ligand TGF-*β*1, including a number of pro-inflammatory outputs [48]. Such transcriptional profiles are being gathered for a variety of epilepsy models by the Consortium for Epilepsy Microarray (Raymond Dingledine, personal communication), and used to define the common set of genes that are modulated across different models of epilepsy, as well as clarify interacting mechanisms and beneficial/detrimental effects. To demonstrate the utility of this approach, we reanalyzed our previous array data from rats treated with albumin, focusing on a subset of genes identified as “core GC responsive genes” by the Microarray Consortium (Figure 1). This allowed us to delineate the numerous transcriptional intersections between stress and our epilepsy model. Of particular note, in context of the mechanisms discussed in this paper, is synergistic modulation of pro-inflammatory cytokine pathways by both albumin treatment and GCs, including chemokine (C-C motif) ligand 2 (Ccl2), interleukin-6 (Il6), tumor necrosis factor (Tnf), and interleukin-1 beta (Il1b). We look forward to future use of these microarray resources, which will elucidate the common pathways in various types of epileptogenesis and allow for nuanced analysis of exacerbating risk factors, such as stress.

## 7. Conclusions

Stress may create vulnerability to epilepsy prior to etiological incidents, as well as exacerbate epileptogenesis following traumatic injury. While potential effects of stress on neural injury are well understood, the ways in which early life stress may create vulnerability for epilepsy, particularly in regard to possible roles for white matter, represent an unknown frontier for future research. While seizures continue to be the defining aspect of epilepsy, nuanced and global analysis of the complex events that occur during epileptogenesis may offer greater insight into the progression of, and possible therapeutic interventions against, epilepsy.
